# Empathy in Medical Education: From Stigma to Understanding Addiction and Improving the Quality of Life of People with Substance Use Disorders

**DOI:** 10.1192/j.eurpsy.2026.10901

**Published:** 2026-07-31

**Authors:** S. T. Bitri, E. K. Bimbashi, E. Sotiri, E. Puca, N. Xhabija

**Affiliations:** 1 Pharmacology And Therapeutics, Western Balkans University, Tirana, Albania; 2 Addiction Medicine, American Hospital; 3 Faculty Of Social Sciences, University of Tirana; 4 Psychiatry; 5 Endocrinology; 6 Cardiology, American Hospital , Tirana, Albania

## Abstract

**Introduction:**

Substance Use Disorders (SUDs) represent a persistent global psychiatric and public health burden, often exacerbated by stigma, fragmented care pathways, and limited integration of psychosocial dimensions in treatment. Traditional biomedical approaches frequently overlook the central role of empathy, which is fundamental in psychiatric practice. Empathy allows clinicians to reframe addiction beyond moral or stigmatizing narratives, to recognize patients’ lived experiences, and to incorporate dimensions such as pleasure and quality of life (QoL) into recovery processes. Despite its importance, empathy remains insufficiently emphasized in undergraduate and postgraduate medical education.

**Objectives:**

This review explores how empathy-centered education during medical school and psychiatric residency can shape clinician attitudes, strengthen therapeutic alliance, and contribute to better recovery trajectories and QoL outcomes for individuals with SUDs.

**Methods:**

A narrative review of peer-reviewed literature was conducted, focusing on medical education, residency training, and addiction psychiatry. Educational strategies reviewed included reflective practice sessions, patient storytelling, exposure to recovery narratives, supervised empathy workshops, and integration of structured curricula promoting person-centered care.

**Results:**

Evidence shows that empathy-focused training improves clinical competence, communication, and stigma reduction. Medical students and residents exposed to empathy-based teaching reported enhanced motivation for compassionate practice and greater willingness to address complex psychosocial factors. Addressing pleasure as part of recovery, when approached empathetically, was associated with improved patient engagement, adherence, and QoL. Conversely, lack of empathy correlated with patient dissatisfaction, disengagement, and weaker therapeutic alliances.

**Conclusions:**

Embedding empathy training across all stages of medical and psychiatric education is essential to bridge the gap between technical knowledge and humanistic care. Empathy not only mitigates stigma but also reframes addiction as a multifaceted condition, supporting recovery, resilience, and enhanced QoL for patients with SUDs.

**Keywords:**

Empathy, Medical Education, Residency, Addiction, Quality of Life

**Disclosure of Interest:**

None Declared

